# RPI-SE: a stacking ensemble learning framework for ncRNA-protein interactions prediction using sequence information

**DOI:** 10.1186/s12859-020-3406-0

**Published:** 2020-02-18

**Authors:** Hai-Cheng Yi, Zhu-Hong You, Mei-Neng Wang, Zhen-Hao Guo, Yan-Bin Wang, Ji-Ren Zhou

**Affiliations:** 1 Xinjiang Laboratory of Minority Speech and Language Information Processing, Xinjiang Technical Institutes of Physics and Chemistry, Chinese Academy of Sciences, Urumqi, 830011 China; 2 University of Chinese Academy of Sciences, Beijing, 100049 China; 3 School of Mathematics and Computer Science, Yichun University, Yichun, 336000 China

**Keywords:** Sequence analysis, RNA-protein interaction, ncRNA, Ensemble learning, Position weight matrix, Legendre moments

## Abstract

**Background:**

The interactions between non-coding RNAs (ncRNA) and proteins play an essential role in many biological processes. Several high-throughput experimental methods have been applied to detect ncRNA-protein interactions. However, these methods are time-consuming and expensive. Accurate and efficient computational methods can assist and accelerate the study of ncRNA-protein interactions.

**Results:**

In this work, we develop a stacking ensemble computational framework, RPI-SE, for effectively predicting ncRNA-protein interactions. More specifically, to fully exploit protein and RNA sequence feature, Position Weight Matrix combined with Legendre Moments is applied to obtain protein evolutionary information. Meanwhile, *k*-mer sparse matrix is employed to extract efficient feature of ncRNA sequences. Finally, an ensemble learning framework integrated different types of base classifier is developed to predict ncRNA-protein interactions using these discriminative features. The accuracy and robustness of RPI-SE was evaluated on three benchmark data sets under five-fold cross-validation and compared with other state-of-the-art methods.

**Conclusions:**

The results demonstrate that RPI-SE is competent for ncRNA-protein interactions prediction task with high accuracy and robustness. It’s anticipated that this work can provide a computational prediction tool to advance ncRNA-protein interactions related biomedical research.

## Background

Protein is the main bearer of cellular activities. However, only a small fraction of the *Human* genome (about 2%) contains protein-coding genes [1]. The remaining 98% of the genes are mainly responsible for regulation, that is, they are involved in controlling when and where genes are expressed and activated [2]. This part of the huge genome produces RNA molecules that vary in size, structure, and function. They are called non-coding RNAs (ncRNA) [3]. Different types of non-coding RNA interact with proteins in different ways. NcRNA can be divided into several categories, which are widely present in most cells and are vital in life activities. And there are some ncRNAs that play a role in specific species. Highly conserved ncRNAs are considered molecular fossils and functional redundancy in the RNA world, and they have been found to act as structural or regulatory molecules involved in the complex flow of life information from DNA to proteins [4].

NcRNA-Protein interactions (ncRPIs) play an essential role in many biological functions. Many ncRNAs play a regulatory role in DNA replication, translation, RNA splicing, and gene expression (such as trans-acting and cis-acting), genome defense and so on [5–7]. Meanwhile, a variety of diseases can be caused by mutations or imbalances in the composition of ncRNAs in the body, such as cancer [8], Prader-Wills syndrome [9], autism [10], Alzheimer’s disease [11], cartilage-hair hypoplasia [12], hearing loss [13]. Because the role of ncRNAs usually depends on binding to specific proteins, identifying the protein molecules that bind to specific ncRNAs is the key to studying the function and mechanism of ncRPIs. Thanks to the Human Genome Project, research in the life sciences has entered the era of post-genomics. The application of various advanced high-throughput experimental methods has generated and accumulated huge amounts of data that are in urgent need of analysis. There is already a gap between the known ncRNAs and their interactions.

High-throughput methods are valuable but time-consuming and expensive. In recent years, there have been extensive research on computational prediction of proteins-RNAs interactions (RPIs) [14–18]. Pancaldi et al. applied both Random Forest (RF) and Support Vector Machine (SVM) model for RPIs prediction, using more than 100 different functional and physical features, such as genomic context, structure or localization, experimental translation and so on [19]. Muppirala et al. introduced a mothed named RPISeq, which also used RF and SVM classifiers based on primary sequences information [20]. In 2013, Lu et al. trained different types Fisher linear discriminant model using the information of hydrogen bonding propensities, the secondary structure and Van der Waals of long ncRNAs and proteins [14]. Suresh et al. presented RPI-Pred, a computational approach based on SVM to predict RPIs by using both high-order structure information [21]. Recently, Cirillo et al. introduced Global Score for protein-RNA interactions prediction. The main contribution of this method is to integrate the local characteristics of protein and RNA structures into the overall binding tendency, and calibrate it based on high-throughput data [22]. Pan et al. put forward a model combined stacking autoencoder with random forest classifiers named IPMiner, archived great prediction performance of ncRPIs [23]. As can be seen, both efficient feature extraction and machine learning model are important to achieve great predictive performance in this domain.

In our previous work, we presented a deep learning stacked autoencoder network based framework to predict ncRNA-protein interactions, named RPI-SAN. The main contribution of RPI-SAN is the application of deep stacked autoencoder to obtain efficient hidden representation of RNA and protein sequence information [18, 24]. Deep learning shows excellent ability with large-scale data support in many fields, however, ncRPIs data sets generally don’t have large scales, thus it’s not very suitable or urgent need for deep learning methods. Previous research confirmed that in ncRPIs prediction task, tree-based model and SVM model can work well, and sequences contain enough information for predicting ncRPIs [25, 26]. Traditional machine learning techniques have the potential to be explored for accuracy and interpretability in small sample learning tasks, especially ncRNA-protein interactions prediction task.

**Fig. 1:**
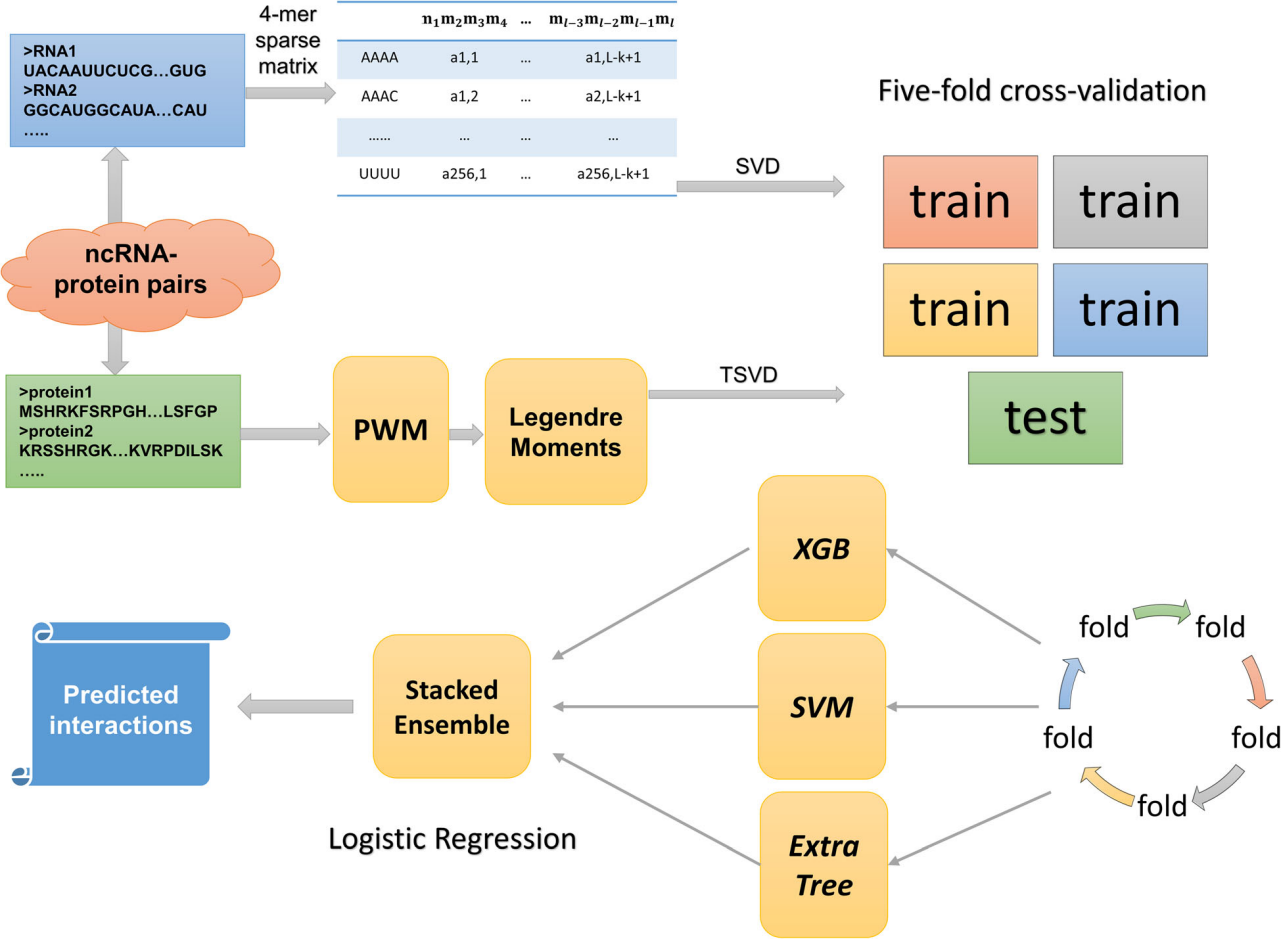
The flowchart of the proposed RPI-SE

To this end, we propose a stacking ensemble based computational model, RPI-SE, by integrating Gradient Boosting Decision Tree (GBDT, implemented by XGBoost) [27], SVM [28, 29] and Extremely randomized Trees [30] (ExtraTree) algorithms to predict ncRNA-protein interactions. Specifically, *k*-mer sparse matrix is used to exploit the sequence information of RNA, which retains not only the nucleic acid components, but also the sequence order information [18, 31, 32]. Meanwhile, the Legendre Moments (LMs) descriptor is applied to convert the information contained in a the Position Weight Matrix (PWM) [33, 34] in a feature vector, which can retain the evolutionary information contained in amino acid sequences corresponding to physicochemical properties. And the Singular Value Decomposition (SVD) [35] is further applied to reduce the dimension of vectors. Then, these evolutionary features are used to train three base predictors include GBDT, SVM and ExtraTree. Finally, stacking ensemble is adopted to integrate these base predictors. To thoroughly verify the performance, the RPI-SE is evaluated on three benchmark data sets under five-fold cross-validation, including RPI369 [20], RPI488 [23] and RPI1807 [21], and compared with other methods, including RPISeq-RF [20], RPI-Pred [21], lncPro [14], IPMiner [23] and RPI-SAN [18]. The experimental results demonstrate that RPI-SE is competent for ncRPIs prediction task, obtained predictive performance with high accuracy and robustness. The workflow of the proposed method is shown in Fig. 1.

## Results

In this work, we proposed a stacking ensemble based computational model to predict ncRNA-protein interactions, called RPI-SE, which integrated XGBoost, SVM and ExtraTree algorithms and using high efficiency features. Above all, we evaluated RPI-SE’s predictive performance of RNA-protein interactions on benchmark data sets. Moreover, we compare RPI-SE with other computational methods on different data sets, including RPI488, RPI369 and RPI1807. Furthermore, the performance of different integration strategies has also been analyzed. The evaluation indicators used in the assessment include accuracy (Acc), true negative rate (TNR), true positive rate (TPR), positive predictive value (PPV), Matthews Correlation Coefficient (MCC) and the Area Under the Receiver Operating Characteristic curve (AUC) are also adopted to evaluate the performance of RPI-SE.

### Evaluate RPI-SE’s performance of RNA-protein interactions prediction

To evaluate RPI-SE’s ability of predicting RNA-protein interactions, the RPI-SE is carried out on RPI369 data set under five-fold cross-validation. The Table 1 shows the results of five-fold cross-validation of RPI-SE on the RPI369 data set. Meanwhile, a comparison of the results of individual base classifiers and stack integration is shown in Table 2. Certainly, the same experiments were performed on RPI488 and RPI1807 data sets, and their results are reported in Additional file 1.

**Table 1:** The five-fold cross-validation performance on RPI369 data set

Fold set	Acc (%)	TPR (%)	TNR (%)	PPV (%)	MCC (%)
1	90.28	86.42	95.89	84.51	81.03
2	88.19	82.56	97.26	78.87	77.61
3	88.19	84.15	94.52	81.69	76.95
4	87.41	81.40	97.22	77.46	76.27
5	88.11	83.95	94.44	81.69	76.81
Average	88.44 ± 1.08	83.69 ± 1.88	95.87 ± 1.38	80.85 ± 2.75	77.73 ± 1.90

**Table 2:** Performance of individual predictors and RPI-SE on RPI369 data set

Predictors	Acc(%)	TPR(%)	TNR(%)	PPV(%)	MCC(%)
XGBoost	84.54	81.45	90.08	78.87	69.51
SVM	75.63	72.50	83.49	67.61	51.86
ExtraTree	68.66	67.65	72.74	64.51	37.57
RPI-SE	**88.44**	**83.69**	**95.87**	**80.85**	**77.73**

The boldface indicates this measure performance is the best among the compared methods

**Fig. 2:**
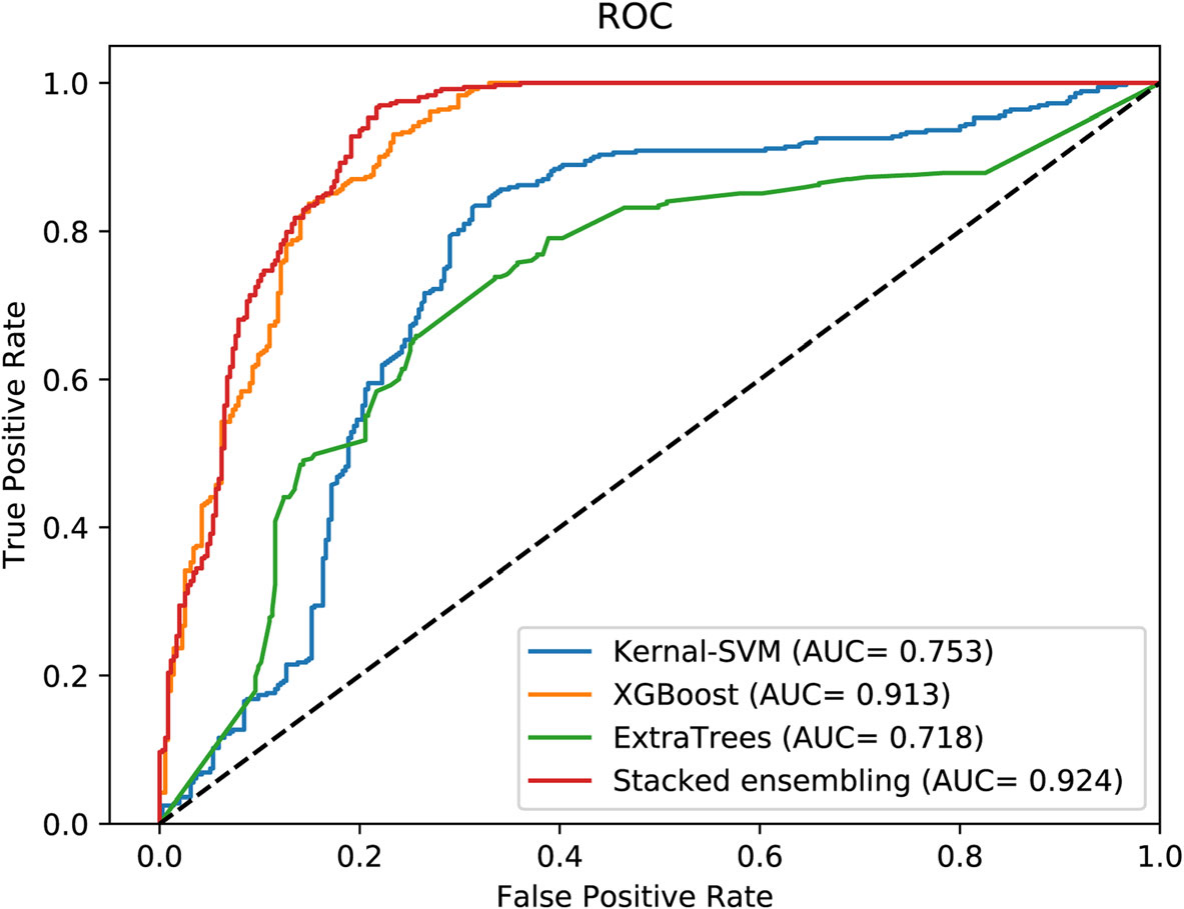
The performance of RPI-SE and contrast methods on RPI369

Under five-fold cross-validation, RPI-SE performs much better than compared methods on RPI369 data set. From Table 2, RPI-SE performs an accuracy of 88.44%, a TPR of 83.69%, a TNR of 95.87%, a PPV of 80.85%, an MCC of 77.73% and as shown in Fig. 2, RPI-SE performed an AUC 0.924. It’s the best of the four comparison predictors. XGBoost achieves an accuracy of 84.54%, a TPR of 81.45%, a TNR of 90.08%, a PPV of 78.87% and MCC of 69.51%. It is the best performing base classifier. The accuracy, TPR, TNR, PPV and MCC of kernel SVM are 75.3, 72.50, 83.49, 67.61 and 51.86% and those of ExtraTree are 68.66, 67.65, 72.74, 64.51% and only 37.57%. The experimental results demonstrate our model is suitable for RNA-protein interaction prediction.

### Comparison between different integrated learning strategies

**Fig. 3:**
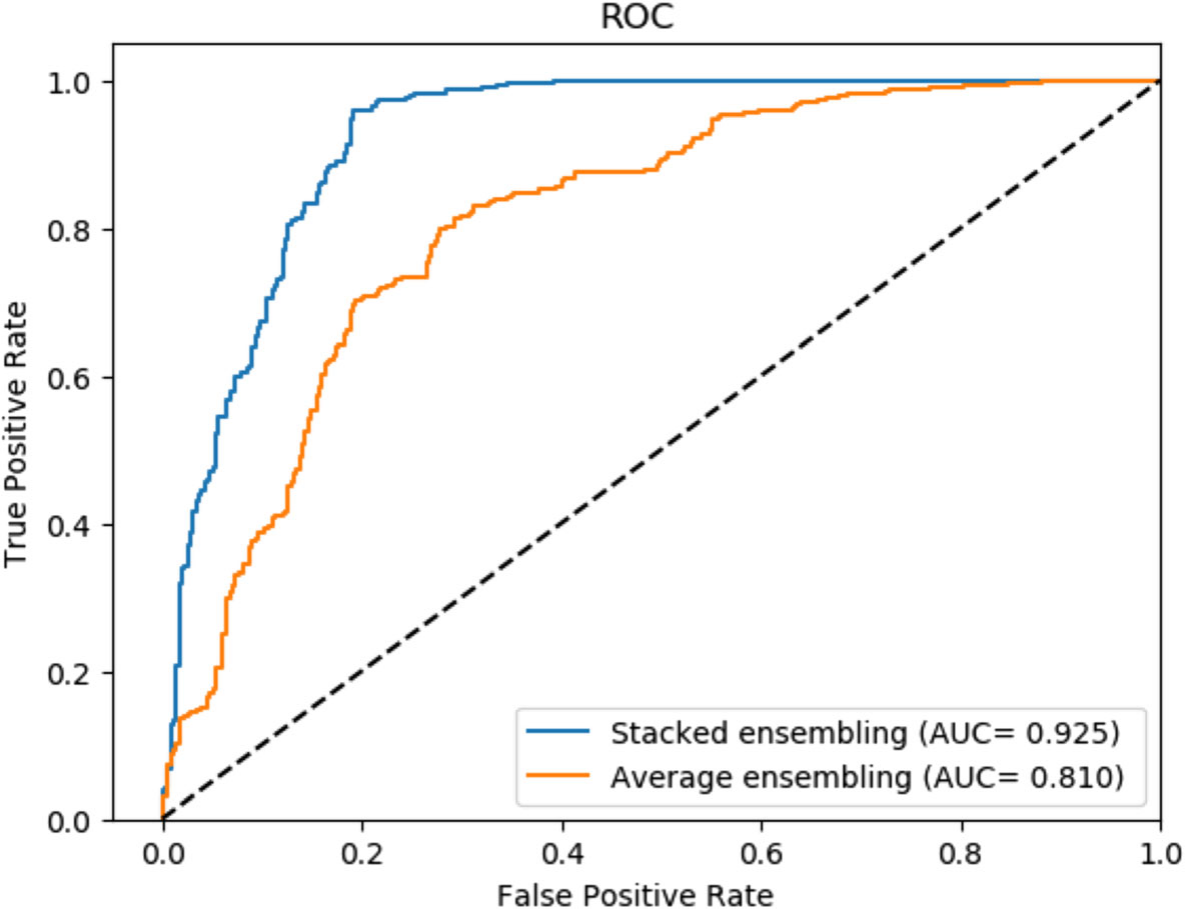
Comparison of different integration strategies

To demonstrate the performance improvement of integration strategies, we compared stacked ensemble with base predictors and general averaged ensemble strategies on RPI369. Stacked ensemble is implemented by a Logistic Regression function. Logistic regression automatically learns respective weights for the three base predictors, including XGBoost, SVM and ExtraTree. As Fig. 3 shows, stacked ensemble archived an AUC of 0.925, better than averaged ensemble method and three base classifiers. Experimental results prove that the stacked integration strategy improves the performance of the prediction framework and is more powerful and flexible than the averaged integration strategy.

### Compared with other state-of-the-art methods

We further compared RPI-SE with other computational methods under same conditions. The contrast methods include IPMiner [23], lncPro [14], RPISeq-RF [20], and RPI-SAN.

**Fig. 4:**
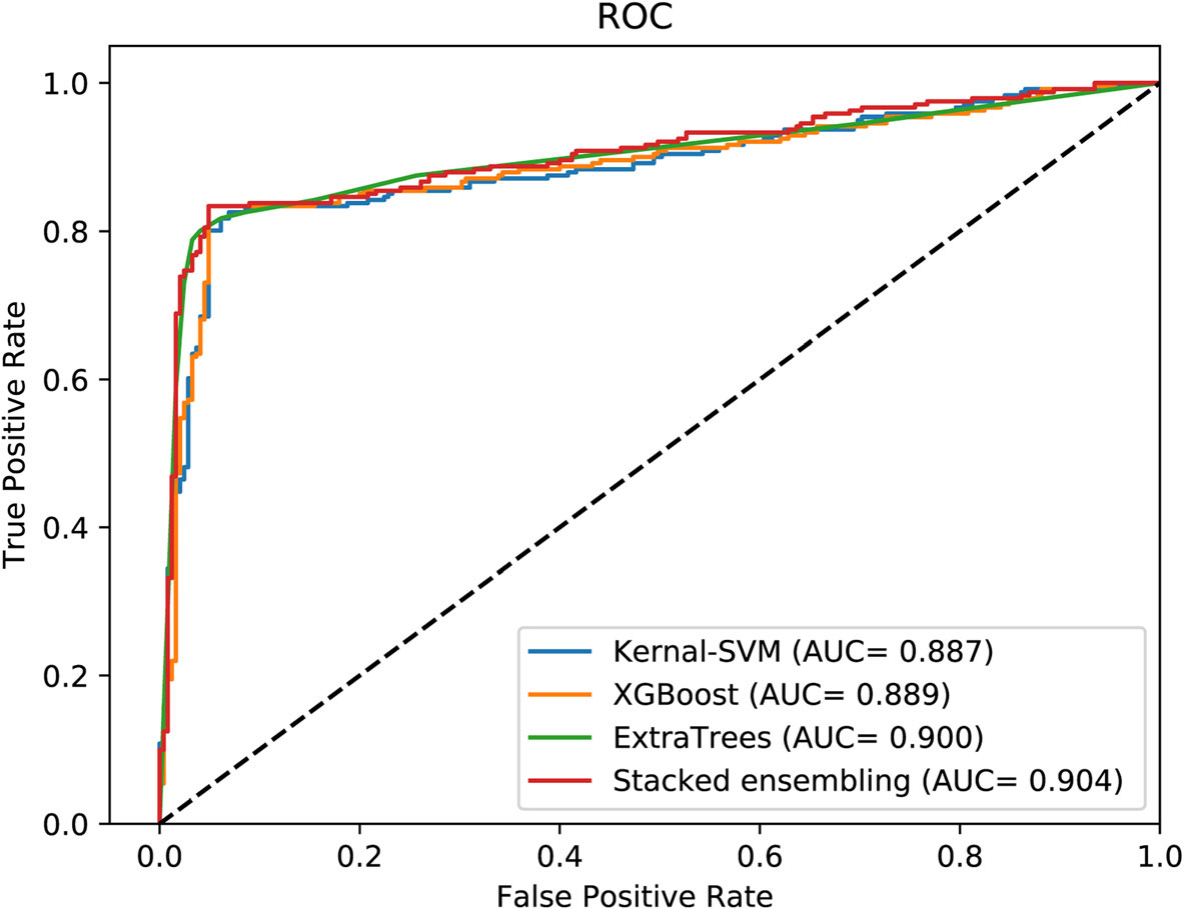
The performance of RPI-SE and contrast methods on data set RPI488

**Fig. 5:**
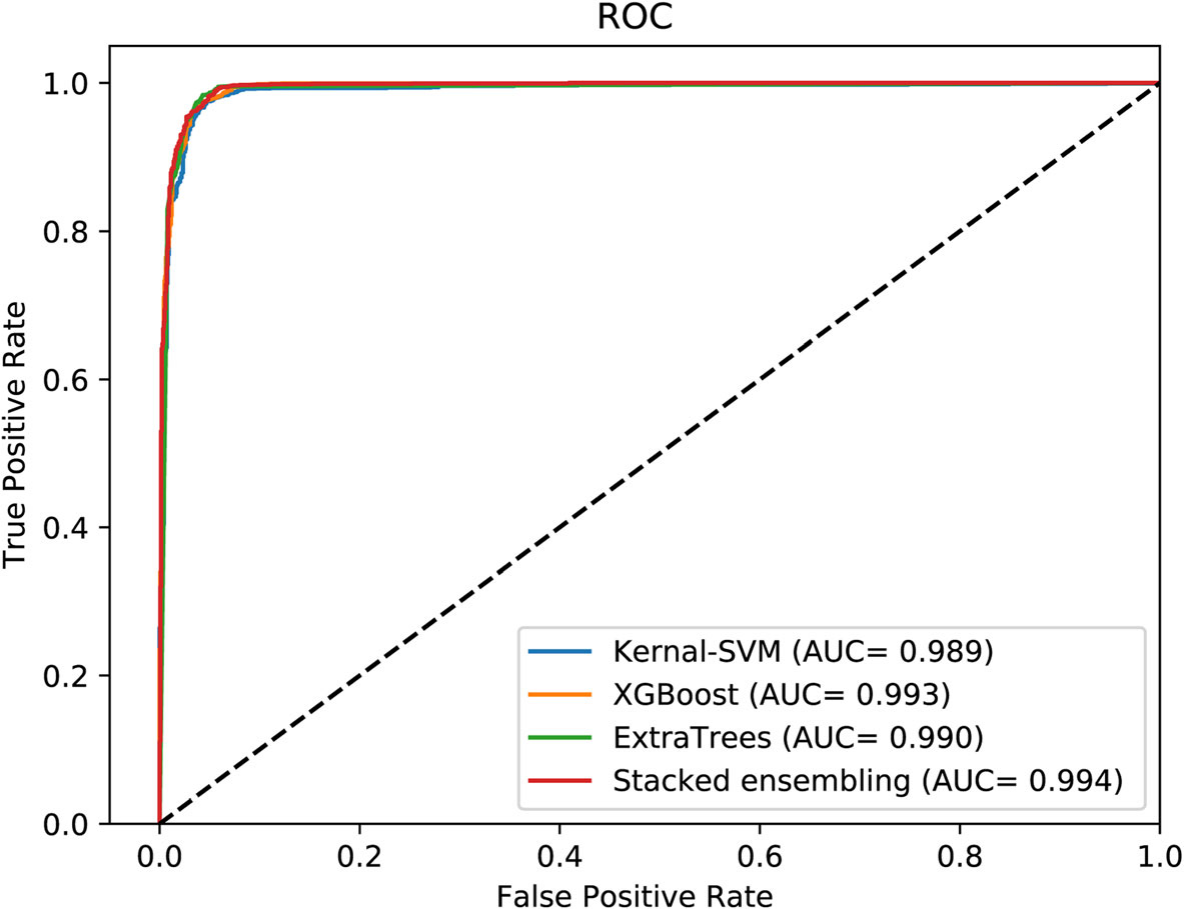
The performance of RPI-SE and contrast methods on data set RPI1807

As Table 3 shows, on RPI369 data set, RPI-SE is obviously better than other methods, with an accuracy of 88.44%, a TPR of 83.69%, a TNR of 95.87%, a PPV of 80.85%, an MCC of 77.73% and AUC of 0.924 (shown in Fig. 2). RPI-SE increased the accuracy, TPR, TNR, PPV, MCC, and AUC by more than 13.2, 10, 16.7, 9.5, 27 and 15%, respectively. For RPI488 data set, RPI-SE also obtained acceptable performance (as AUC shown in Fig. 4), with an accuracy of 89.3%, better than other comparison methods but only closed to RPI-SAN. As shown in Table 3 and Fig. 5, on the data set RPI1807, the results of all the methods are close, with an accuracy rate of over 96%. RPI-SE attains a high accuracy of 96.86%.

**Table 3:** Compared RPI-SE with other computational methods on RPI369, RPI488 and RPI1807 data sets

Data sets	Methods	Acc(%)	TPR(%)	TNR(%)	PPV(%)	MCC(%)	AUC
RPI369	IPMiner	75.2	73.5	79.1	71.3	50.7	0.773
	RPISeq-RF	70.4	70.5	70.2	70.7	40.9	0.767
	lncPro	70.4	70.8	69.6	71.3	40.9	0.740
	RPI-SAN	74.9	74.1	78.7	71.7	50.4	0.778
	RPI-SE	88.44	83.69	95.87	80.85	77.73	0.924
RPI488	IPMiner	89.1	93.9	83.1	94.5	78.4	0.914
	RPISeq-RF	88.0	92.6	82.2	93.2	76.2	0.903
	lncPro	87.0	90.0	82.7	91.0	74.0	0.901
	RPI-SAN	89.7	94.3	83.7	95.2	79.3	0.920
	RPI-SE	89.30	94.49	83.48	95.15	79.31	0.904
RPI1807	IPMiner	98.6	98.2	99.3	97.8	97.2	0.998
	RPISeq-RF	97.3	96.8	98.4	96.0	94.6	0.996
	lncPro	96.9	96.5	98.1	95.5	93.8	0.994
	RPI-SAN	96.1	93.6	99.9	91.4	92.4	0.999
	RPI-SE	96.86	96.71	97.69	95.83	93.65	0.994

## Discussion

RPI-SE is composed of three basic predictors, XGBoost classifier, SVM classifier with RBF kernel, and ExtraTree classifier. Different classifiers have different adaptability to the data. XGBoost has advantages in accuracy and TPR, while SVM has advantages in stability. At the same time, basic classifiers have their own disadvantages. It is necessary to integrate them for best performance. The degree to which the stacking strategy improves the final prediction performance is different. When the difference between the classifiers is greater, stacking integration is more effective. The RPI488 and RPI1807 data sets have stronger correlations, so the base predictors have more consistent output on these two data sets, and the stacking ensemble improves the performance of the prediction framework less on these two data sets. RPI-SE uses PWM to convert a protein sequence into a probabilistic description, which requires that the sequence length be greater than 50. Therefore, sequences less than 50 in length were removed. The performance of machine learning models is highly dependent on the parameter set, while the model parameters of RPI-SE are only adjusted on the RPI369 data set, which makes it not perform optimally on the other two data sets. It uses only simple machine learning models and integration strategies to achieve results that are close to or better than the most advanced models. These results proved it is an acceptable methodological innovation in terms of simplicity and efficiency.

## Conclusion

In this research, we put forward a stacking ensemble computational method, RPI-SE, integrated three individual models, including XGBoost, SVM and ExtraTree, to predict ncRNA-protein interactions using sequence information. PWM and *k*-mer sparse matrix were employed to fully mine efficient features from protein and RNA sequences. The presented method gained a great performance on benchmark data sets. Experimental results prove that the proposed method can accurately and efficiently predict potential ncRNA-protein interactions. RPI-SE uses only simple machine learning models and ensemble learning strategy, and has the advantages of simplicity and interpretability. Meanwhile, RPI-SE has better performance on small data sets, which is in line with the limited scale of the ncRNA-protein interaction data. Although deep learning has been widely adopted in many fields, there is still plenty valuable work worth doing. As a general machine learning model, RPI-SE can perform ncRPI predictions more conveniently and rapidly than complex deep learning models, which can provide useful guidance for ncRPI related biomedical research.

## Methods

### Data sets

Three benchmark data sets from the previous research, including RPI369, RPI488 and RPI1807, are used to evaluate the performance of RPI-SE. The RPI369 is a non-redundant data set without ribosomal proteins or ribosomal RNAs, from PRIDB [36], which is a comprehensive database calculated from the Protein Data Bank (PDB) [37] of protein-RNA complexes. It includes a total of 332 RNA chains and 338 protein chains, and 369 positive interactive pairs. The RPI488 is a non-redundant lncRNA-protein interactions data set, has 245 negative samples and 243 positive samples [38–40]. The RPI1807 data set includes 1078 RNA and 1807 protein chains. And the number of positive and negative samples is 1807 and 1436, contains 493 RNA and 1436 protein chains. The details of the data sets used in this work are shown in Table 4.

**Table 4:** The details of the RNA-protein interaction data sets

Data set	Interaction pairs	# of proteins	# of RNAs
RPI369	369	338	332
RPI488	243	25	247
RPI1807	1807	1807	1078

### The ncRNA and protein sequences representation

To fully explore the evolutionary features of ncRNA and protein sequences, *k*-mer sparse matrix and position weight matrix are used to represent RNA and protein sequences, respectively. RNA sequence were represented by the *k*-mer sparse matrix [31]. From beginning to end, it scans each RNA sequence (A, C, G, U) with a *k* nucleotides window, move one nucleotide at a time. Suppose a RNA sequence with length of *L*, there are 4^*k*^ different possible *k*-mers and *L* − *k* + 1 steps.

As shown in Table 5, the dimension of the corresponding *k*-mer sparse matrix *M* is 4^*k*^ × (*L* − *k* + 1). When *m*_*j*_*m*_*j* + 1_*m*_*j* + 2_*m*_*j* + 3_ are same to the *i*_th_ *k*-mer among 4^*k*^ different *k*-mers, set the element *a*_*ij*_ to 1.

**Table 5:** *K*-mer sparse matrix representation of RNA sequence

	R_1_R_2_R_3_R_4_	R_2_R_3_R_4_R_5_	…	R_L-3_R_L-2_R_L-1_R_L_
AAAA	a_11_	a_12_	…	a_1,L-k + 1_
AAAC	a_21_	a_22_	…	a_2,L-k + 1_
AACA	a_31_	a_32_	…	a_3,L-k + 1_
…	…	…	…	…
UUUU	a_256,1_	a_256,2_	…	a_256,L-k + 1_

The *k*-mer sparse matrix *M* can be defined as follows and the *k* is set to 4 for RNA sequence.

1 $$ \mathrm{M}={\left({\mathrm{a}}_{\mathrm{ij}}\right)}_{4^{\mathrm{k}}}\times \left(\mathrm{L}-\mathrm{k}+1\right) $$

2 $$ {a}_{ij}=\left\{\begin{array}{c}1, if\ {\mathrm{m}}_{\mathrm{j}}{\mathrm{m}}_{\mathrm{j}+1}{\mathrm{m}}_{\mathrm{j}+2}{\mathrm{m}}_{\mathrm{j}+3}=k- mer(i)\\ {}0, else\end{array}\right. $$

Moreover, the SVD is adopted to reduce *M* into a 1 × 256 feature vector.

In consideration of the different structures between RNA and protein sequences have, we employed a more biological method for protein sequences to contain biological evolution information, the position weight matrix (PWM), which is a widely used representation of motifs in biological sequences, to convert it. A PWM has one row for each symbol of the alphabet and 20 rows for amino acids in protein sequences. The PWM of a protein sequence with length of *l* can defined as follow:

3 $$ PWM=\left[\begin{array}{c}{w}_{1,1},{w}_{1,2},\dots, {w}_{1,20}\\ {}{w}_{2,1},{w}_{2,2},\dots, {w}_{2,20}\\ {}\vdots \vdots \vdots \vdots \\ {}{w}_{l,1},{w}_{l,2},\dots, {w}_{l,20}\end{array}\right] $$

In practice, both the Position-Specific Iterated BLAST (PSI-BLAST) tool and against database *SwissProt* can be freely downloaded from http://blast.ncbi.nlm.nih.gov/Blast.cgi. And we set err-value to 0.001, set the value of iteration to 3.

Then we extracted Legendre Moment (LMs) [41] feature vectors from the PWM of protein sequence. LMs can exploit eigenvectors of a matrix without losing information, in which the Legendre polynomial is adopted as the kernel function. It is a type of class orthogonal moment, which is widely used in image analysis and pattern recognition.

The 2-D Legendre moments of order (*m*, *n*), with image intensity function *f* (*x*; *y*), are defined as:

4 $$ {L}_{mn}={\mu}_{mn}{\int}_{-1}^1{\int}_{-1}^1{V}_m(x){V}_n(x)f\left(x,y\right) dxdy $$

where *m, n* = 0, 1, 2..., *μ*_*mn*_ = (2 *m* + 1)(2*n* + 1)/4, and the *m*_th_ order LMs is given by:

5 $$ {V}_m(x)=\frac{1}{2^mm!}\frac{d_m}{d_{x^m}}{\left({x}^2-1\right)}^m $$

which has the following orthogonality, where ϑ_*mn*_ represents the Kronecker function.:

6 $$ {\int}_{-1}^1{\mathrm{V}}_{\mathrm{m}}\left(\mathrm{x}\right){\mathrm{V}}_{\mathrm{n}}\left(\mathrm{x}\right)=\frac{2}{2\mathrm{m}+1}{\upvartheta}_{mn} $$

Hence, a matrix of R × S elements with function *f* (*i, j*) can be indicated in discrete form as follow:

7 $$ {L}_{mn}={\mu}_{mn}{\sum}_{i=1}^R{\sum}_{j=1}^S{h}_{mn}\left(x,y\right)f\left(x,y\right) $$

For the Legendre polynomials,

8 $$ \int {V}_m(x) dx=\raisebox{1ex}{${V}_{m+1}(x)-{V}_{m-1}(x)$}\!\left/ \!\raisebox{-1ex}{$2m+1$}\right.,x\in \left[-1,1\right] $$

So, according to the above formula, the accuracy expression can be defined as follows.

9 $$ {L}_{mn}={\mu}_{mn}{\sum}_{i=0}^{R-1}{\sum}_{j=0}^{S-1}\frac{\Delta  \left(m,x\right)}{2m+1}\times \frac{\Delta  \left(n,y\right)}{2n+1} $$

10 $$ \Delta  \left(\mathrm{p},\mathrm{t}\right)={V}_{p+1}\left(t+\frac{\Delta  t}{2}\right)-{V}_{p-1}\left(t+\frac{\Delta  t}{2}\right)-{V}_{p+1}\left(t-\frac{\Delta  t}{2}\right)+{V}_{p-1}\left(t-\frac{\Delta  t}{2}\right) $$

Therefore, a PWM of a target protein sequence will be converted into a 1 × 676 feature vector by using LMs. The truncated SVD was further employed to reduce the influence of noise and retain the principal features. Truncated SVD is very similar to principal component analysis (PCA), but differs in that it works on sample matrices directly instead of their covariance matrices. Contrary to PCA, this estimator does not center the data before computing the singular value decomposition. This means it can work with sparse matrices efficiently. When truncated SVD is applied to term-document matrices, it is known as Latent Semantic Analysis [42]. The feature vectors of the protein will be reduced to 500 dimensions. Finally, each pair of ncRNA-protein contains 1 × 756 conjoined feature vector.

### To-be-integrated machine learning classifier

Three kinds of machine learning classifiers are used as to-be-integrated base classifiers, including GBDT [27], SVM [28, 29] and ExtraTree [30].

XGBoost is a scalable end-to-end tree boosting model implementation, which is a great sparsity-aware approach for sparse data and weighted quantile sketch for approximate tree learning. Traditional GBDT only uses first-order derivative information when optimizing. XGBoost performs second-order Taylor expansion for cost function, and uses first and second derivatives. It adds a regularization term in the cost function to control the complexity of the model. A regular term contains the number of leaf nodes of a tree, and the sum of squares of the L2 modules of score on each leaf node. From the Bias-variance tradeoff point of view, the regular term reduces the variance of the model, making the learning model simpler and preventing over fitting, which is also a characteristic of its superior to the traditional GBDT. After iteration, XGBoost multiplied the weight of the leaf node, mainly to weaken the impact of each tree, and let the behind have a larger learning space. XGBoost draws on the practice of random forest and supports column sampling, which not only reduces over-fitting but also reduces calculations. A parallel approximate histogram algorithm is also proposed to generate candidate segmentation points efficiently. XGBoost’s objective function can be defined as follows:

11 $$ \mathrm{Obj}={\sum}_{i=1}^nl\left({y}_i,{\hat{y}}_i\right)+{\sum}_{k=1}^K\Omega \left({f}_k\right) $$

12 $$ \Omega \left({f}_t\right)=\upgamma \mathrm{T}+\frac{\uplambda}{2}{\sum}_{j=1}^T{w}_j^2 $$

Here, *l* is a differentiable convex loss function that measures the difference between the prediction ${\hat{y}}_i$ and the target *y*_*i*_. The regular term controls the complexity of the model, including the number of leaf nodes T and the *l*_2_ modulus square of the leaf score.

SVM constructs a hyperplane or a series of hyperplanes in a high-dimensional or infinite-dimensional space that can be used for classification, regression, or other tasks. Intuitively, by using a hyperplane to achieve a good segmentation, it is possible to maximize the distance between the closest training data points (function margins) in any class. This is usually due to a larger margin. The advantages of support vector machines are: It’s very efficient in high dimensional space. Even if the data dimension is larger than the sample size, it is still valid. The subset of training sets is used in support vectors, so it is also efficient in memory utilization. The disadvantages of support vector machines include: If the number of features is much larger than the number of samples, it is necessary to avoid overfitting when selecting kernel functions.

Suppose the labeled training data [(*x*_*i*_, *y*_*i*_), *i* = 1, 2, 3…, *n*, *y*_*i*_ = (− 1, 1), *x*_*i*_∈ R]. and the separating hyperplane is: (*w(x) + b*) = 0. In the linear separable situation, the SVM maximized the margin by minimizing ‖*w*‖^2^/2 subject to looking for the separating hyperplane as following constraint:

13 $$ {y}_i\left({w}_{x_i}+\mathrm{b}\right)\ge 1,\forall {x}_i $$

In the linear non-separable situation, we can find the optimal separating hyperplane by introducing slack variables: ξ_*i*_, *i* = 1, 2..., *n* and user-adjustable parameter C, then minimizing:

14 $$ {\left\Vert w\right\Vert}^2/2+C{\sum}_{i=1}^n{\upxi}_i,{\upxi}_i\ge 0,\forall {x}_i $$

15 $$ {y}_i\left({w}_{x_i}+\mathrm{b}\right)\ge 1-{\upxi}_i,{\upxi}_i\ge 0,\forall {x}_i $$

Radial Basis Function (RBF) kernel is adopted in this experiment, which can be defined as:

16 $$ f\left(\mathrm{x}\right)={e}^{-\gamma {\left\Vert x-{x}^{\prime}\right\Vert}^2} $$

Extremely randomized trees essentially consist of randomizing strongly both attribute and cut-point choice while splitting a tree node. It builds totally randomized trees whose structures are independent of the output values of the learning sample. The strength of the randomization can be tuned to problem specifics by the appropriate choice of a parameter. Randomness in the computation of segmentation points is further enhanced. In a random forest, a random subset of the candidate features is used in a random forest. Unlike a threshold for finding the most regional diversity, the threshold here is randomly generated for each candidate feature and selects the best one of these randomly generated thresholds as a segmentation rule. This method usually reduces the variance of one-point model, while the cost slightly increases the deviation.

### Implementation of stacking ensemble integration strategy

The Logistic Regression (LR) is used as the merge layer to integrate three base classifiers’ output, which can learn the integration weight *w* for each base classifier. The predicted probability value outputs of individual classifiers be the level 0 layer, while successive logistic regression was the level 1. The definition of LR is:

17 $$ {P}_w\left(\pm 1|p\right)=\frac{1}{1+{e}^{-{w}^Tp\left(\pm 1|p\right)}} $$

where the *p* is the level 0 classifiers’ probability outputs and it will degenerate to average strategy when the weight for each individual classifier of logistic regression is judged as the same.

### Performance evaluation indicators

The evaluation of the experiments in this work was performed under five-fold cross-validation. In each validation, all data randomly divides into five equal subsets, four-fold data are used for training, and the rest one-fold is used for testing. There is no overlap between train data and test data. The average performances of five-fold are taken as the final validation performance. The evaluation indicators used in the experiments can be defined as:

18 $$ \mathrm{Acc}=\frac{TN+ TP}{TN+ TP+ FN+ FP} $$

19 $$ \mathrm{TPR}=\frac{TP}{TP+ FN} $$

20 $$ \mathrm{TNR}=\frac{TN}{TN+ FP} $$

21 $$ \mathrm{PPV}=\frac{TP}{TP+ FP} $$

22 $$ \mathrm{MCC}=\frac{TP\times TN- FP\times FN}{\sqrt{\left( TP+ FP\right)\left( TP+ FN\right)\left( TN+ FP\right)\left( TN+ FN\right)}} $$

where *TN*, *TP*, *FN*, and *FP* indicates the number of true negative, true positive, false negative and false positive samples.

## Supplementary information

**Additional file 1:** **Table S1.** The 5-fold cross-validation details on RPI488 dataset. **Table S2.** Performance of individual predictors and RPI-SE on RPI488 dataset. **Table S3.** The 5-fold cross-validation details on RPI1807 dataset. **Table S4.** Performance of individual predictors and RPI-SE on RPI1807 dataset.

## Data Availability

The datasets generated and/or analysed during this study are available under open licenses in the data repository, https://github.com/haichengyi/RPI-SE.

## Abbreviations

Acc: Accuracy
AUC: Area Under the receiver operating characteristic Curve
ExtraTree: Extremely randomized Trees
GBDT: Gradient Boosting Decision Tree
LMs: Legendre Moments
LR: Logistic Regression
MCC: Matthews Correlation Coefficient
ncRNA: non-coding RNA
ncRPI: non-coding RNA-protein interaction
PCA: Principal Component Analysis
PDB: Protein Data Bank
PPV: Positive Predictive Value
PSI-BLAST: Position-Specific Iterated BLAST
PWM: Position Weight Matrix
RF: Random Forest
RPI: RNA-protein interaction
SVD: Singular Value Decomposition
SVM: Support Vector Machine
TNR: True Negative Rate
TPR: True Positive Rate
