# Supplementary material for: RPI-SE: a stacking ensemble learning framework for ncRNA-protein interactions prediction using sequence information
Source: BMC Bioinformatics. 2020 Feb 18;21:60. doi: 10.1186/s12859-020-3406-0 (PMC7029608; doi:10.1186/s12859-020-3406-0)
Supplement: Supplementary file 1 — Additional file 1: Table S1. The 5-fold cross-validation details on RPI488 dataset. Table S2. Performance of individual predictors and RPI-SE on RPI488 dataset. Table S3. The 5-fold cross-validation details on RPI1807 dataset. Table S4. Performance of individual predictors and RPI-SE on RPI1807 dataset. [file 12859_2020_3406_MOESM1_ESM.pdf]

Table S1. The 5-fold cross-validation details on RPI488 dataset.

Fold set	Acc(%)	TPR(%)	TNR(%)	PPV(%)	MCC(%)
**1**	87.76	93.02	81.63	93.88	76.08
**2**	90.72	95.24	85.11	96.00	81.81
**3**	89.69	100.00	80.00	100.00	81.22
**4**	86.60	92.68	79.17	93.88	73.93
**5**	91.75	91.49	91.49	92.00	83.49
**Average**	89.30±2.11	94.49±3.37	83.48±5.02	95.15±3.06	79.31±4.08

Table S2. Performance of individual predictors and RPI-SE on RPI488 dataset.

Predictors	Acc(%)	TPR(%)	TNR(%)	PPV(%)	MCC(%)
**XGBoost**	89.10	94.04	83.48	94.75	78.86
**SVM**	87.86	93.45	81.39	94.34	76.47
**ExtraTree**	86.63	89.33	83.07	90.18	73.53
**RPI-SE**	**89.30**	**94.49**	**83.48**	**95.15**	**79.31**

Table S3. The 5-fold cross-validation details on RPI1807 dataset.

Fold set	Acc(%)	TPR(%)	TNR(%)	PPV(%)	MCC(%)
**1**	96.39	96.63	96.90	95.76	92.69
**2**	96.87	97.45	96.90	96.82	93.66
**3**	96.08	95.82	97.17	94.72	92.07
**4**	96.86	96.13	98.31	95.05	93.65
**5**	98.12	97.50	99.15	96.82	96.19
**Average**	96.86±0.78	96.71±0.76	97.69±1.00	95.83±0.98	93.65±1.57

Table S4. Performance of individual predictors and RPI-SE on RPI1807 dataset.

Predictors	Acc(%)	TPR(%)	TNR(%)	PPV(%)	MCC(%)
**XGBoost**	96.89	97.24	97.18	96.54	93.72
**SVM**	96.52	96.27	97.52	95.27	92.95
**ExtraTree**	97.33	97.05	98.19	96.26	94.60
**RPI-SE**	96.86	96.71	97.69	95.83	93.65
